# Balanced Sparse Model for Tight Frames in Compressed Sensing Magnetic Resonance Imaging

**DOI:** 10.1371/journal.pone.0119584

**Published:** 2015-04-07

**Authors:** Yunsong Liu, Jian-Feng Cai, Zhifang Zhan, Di Guo, Jing Ye, Zhong Chen, Xiaobo Qu

**Affiliations:** 1 Yunsong Liu, Zhifang Zhan, Jing Ye, Zhong Chen, Xiaobo Qu Department of Electronic Science/Fujian Provincial Key Laboratory of Plasma and Magnetic Resonance, Xiamen University, Xiamen, China; 2 Jian-Feng Cai Department of Mathematics, University of Iowa, Iowa City, Iowa, USA; 3 Di Guo School of Computer and Information Engineering, Xiamen University of Technology, Xiamen, China; Wadsworth Center, UNITED STATES

## Abstract

Compressed sensing has shown to be promising to accelerate magnetic resonance imaging. In this new technology, magnetic resonance images are usually reconstructed by enforcing its sparsity in sparse image reconstruction models, including both synthesis and analysis models. The synthesis model assumes that an image is a sparse combination of atom signals while the analysis model assumes that an image is sparse after the application of an analysis operator. Balanced model is a new sparse model that bridges analysis and synthesis models by introducing a penalty term on the distance of frame coefficients to the range of the analysis operator. In this paper, we study the performance of the balanced model in tight frame based compressed sensing magnetic resonance imaging and propose a new efficient numerical algorithm to solve the optimization problem. By tuning the balancing parameter, the new model achieves solutions of three models. It is found that the balanced model has a comparable performance with the analysis model. Besides, both of them achieve better results than the synthesis model no matter what value the balancing parameter is. Experiment shows that our proposed numerical algorithm constrained split augmented Lagrangian shrinkage algorithm for balanced model (C-SALSA-B) converges faster than previously proposed algorithms accelerated proximal algorithm (APG) and alternating directional method of multipliers for balanced model (ADMM-B).

## Introduction

Magnetic resonance imaging (MRI) is an important imaging modality in clinical diagnosis to investigate anatomy and function of the body [1–6]. It is non-radioactive, non-invasive, and has rich contrast information such as T1 and T2. However, the data acquisition speed in MRI is fundamentally limited by physical (gradient amplitude and slew-rate) and physiological (nerve stimulation) constraints [2].

Compressed sensing (CS) MRI has shown its strong ability to reduce the data acquisition time and earned a lot of attentions over the last few years [2, 3, 7–9]. This new technology, denoted as CS-MRI, reduces the number of measurements required by Nyquist sampling criteria and tries to reconstruct an image that is sparse or can be sparsely represented in some transform domains, e.g. wavelets and finite difference [2, 3]. According to the CS theory, under some conditions, the number of required Fourier samples for an *N*-dimensional signal with *S* non-zeroes (sparsity) in some transform domain to be successfully recovered with a dominant probability is governed by *O*(*S*log*N*). This condition is much less than *N* when the interested signal is very sparse (*S* ≪ *N*) [10, 11].

Orthogonal wavelets (orthogonal systems) are usually used in traditional compressed sensing MRI and is simple and effective [2, 12, 13]. However, orthogonal wavelets may lead to blocky artifacts in image reconstruction [14–17]. On the contrary, redundant wavelets, such as X-let [18–24] and others [25–31], can significantly improve the image quality [14–17]. Some of these transforms, e.g. contourlet [18] and patch-based directional wavelet (PBDW) [14], a simplified form of bandelet, have been investigated in CS-MRI and shown advantages on edge reconstruction and noise removal [14, 32]. Besides, researchers have utilized the wavelet coefficients’ structure and significantly improve the image quality in CS-MRI [33, 34]. But how to investigated these property under tight frame wavelet is unsolved and needs careful investigation which is beyond the scope of this paper.

There are two different data models for general signal or image processing, namely, the analysis and synthesis models with different prior assumptions [35, 36]. Elad et al. studied these two models and offered a geometric explanation of the relation between them [35]. Although the synthesis model has attracted more attention than the analysis model in the past, recent studies show that the latter has its own advantage over the former both theoretically [37, 38] and empirically [36]. In the field of CS-MRI, Qu et al. [14, 15] and Yang et al. [39, 40] have modeled their problems as analysis models and achieved satisfying results.

To bridge analysis and synthesis models, Cai et al. [41–44] proposed a balanced model. The balanced model has been applied to image restoration including deblurring, inpainting, and astronomy image reconstruction and solved by a proximal forward-backward splitting (PFBS) algorithm [45]. Furthermore, Shen et al. proposed an accelerated proximal gradient (APG) algorithm to solve the balanced model in image inpainting and deblurring [46] with an accelerating scheme that is much similar to a fast iterative shrinkage-thresholding algorithm (FISTA) [47]. Unlike these two iterative shrinkage algorithms, Xie et al. proposed an alternating direction method of multipliers algorithm to solve the balanced model called ADMM-B in image inpainting and deblurring [48]. By using Sherman-Morrison-Woodbury matrix inversion lemma, their experiments showed the much faster speed of ADMM-B than APG. Another benefit of ADMM-B is that it allows the balancing parameter *β* to change from 0 to +∞ without any influence on the convergence speed.

The motivation of this paper comes from three aspects: 1) a tight frame usually outperforms its corresponding orthogonal transform in CS-MRI, but many researchers in CS-MRI are not aware of the difference between the analysis and synthesis models when tight frame is used; 2) it is still unknown how the performance changes during the transition from the analysis model to the synthesis model in CS-MRI; 3) there is no unified view of which model is better in general, and our observation found that the analysis model always has the best performance in CS-MRI.

The contribution of this paper is two folded. First of all, we will explore the performance of the balanced model for tight frame based CS-MRI, which, to the best of our knowledge, has never been investigated before. We will discuss the impact of the balancing parameter on the reconstruction error. Secondly, we will propose a new efficient numerical algorithm for solving the balanced model. The proposed algorithm has a faster convergence than existing algorithms for the balanced model such as APG [46] and ADMM-B [48]. Besides, the proposed balanced model provides a unified framework to explore the performance of three sparse models in specific applications of CS-MRI, in which case the comparison results are not known yet before.

## Methods

### Ethics Statement

All human images were acquired from healthy subjects under the approval of the Institute Review Board of Xiamen University and written consent was obtained from the participants. The data were analyzed anonymously.

The *k*-space data undersampling in CS-MRI can be formulated as

$$\begin{array}{c} y=UFx+\eta , \end{array}$$ (1)

where ***U*** ∈ ℂ^*M*×*N*^ with *M* < *N* is an undersampling operator, ***F*** ∈ ℂ^*N*×*N*^ represents the discrete Fourier transform, and ***η*** ∈ ℂ^*M*^ is the noise. CS-MRI aims at reconstructing an image ***x*** ∈ ℂ^*N*^ from the undersampled data ***y*** ∈ ℂ^*M*^. This image reconstruction problem is an under-determined linear inverse problem that has infinite solutions. Additional constraints should be introduced to obtain a unique solution that meets the realistic magnetic resonance (MR) image priors.

### Related Work

#### Synthesis model in compressed sensing

According to CS theory [10, 11, 49], a fine reconstruction of (1) is achievable by solving the following ℓ_1_-norm based optimization problem

$$\begin{array}{c} \hat{x}=D\hat{\alpha },\;\;\hat{\alpha }=\arg\min_{\alpha }{∥\alpha ∥}_{1},\;\text{s.t.}\;{∥y-UFD\alpha ∥}_{2}^{2}\leq \sigma^{2}, \end{array}$$ (2)

where ***D*** is a synthesis dictionary, ***α*** is the corresponding coefficient, $\hat{\text{x}}$ is the reconstructed MR image. *σ* ^2^ is related to the noise variance of the measured data. The performance of (2) is governed by

$$\begin{array}{c} ∥\hat{\alpha }{-\alpha ∥}_{2}\leq C_{0}\frac{∥\alpha -\alpha_{S}{∥}_{1}}{\sqrt{S}}+C_{1}\sigma , \end{array}$$ (3)

provided that restricted isometry property (RIP) constant *δ*_2*S*_ of ***UFD*** obeys $\delta_{2S}<\sqrt{2}-1$ [49]. Here ***α***_*S*_ is the best approximation to ***α*** by using at most *S* nonzeroes. To let ***UFD*** satisfy RIP, the undersampling matrix ***U*** is chosen randomly, and, more importantly, the columns of ***D*** should have a small mutual coherence in the sense of a small RIP constant [50]. Equation (3) implies that a good reconstruction can be obtained from (2) if a MR image is a sparse combination of atom signals which are columns of ***D***. Models like (2) that directly solves ***α*** are called a synthesis model.

#### Analysis model in compressed sensing

The analysis model is

$$\begin{array}{c} \hat{x}=\arg\min_{x}{∥\Psi x∥}_{1},\;\text{s.t.}\;{∥y-\mathrm{UF}x∥}_{2}^{2}\leq \sigma^{2} \end{array}$$ (4)

where **Ψ** is an analysis operator to sparsify the image. It is clear that the solution of (4) is an image. The theoretical guarantee of an analysis model becomes

$$\begin{array}{c} ∥\hat{x}{-x∥}_{2}\leq C_{0}\frac{{∥\Psi x-\left(\Psi x\right)}_{S}{∥}_{1}}{\sqrt{S}}+C_{1}\sigma , \end{array}$$ (5)

provided that the rows of **Ψ** form a (tight) frame and ***U*** ***F*** satisfies Ψ*-RIP with constant *δ*_2*S*_ < 0.08 [29]. Note that there is no incoherence restriction on the rows of the analysis operator **Ψ**. The analysis model is also theoretically studied in [38] where the model is called cosparse analysis model.

#### Analysis model versus synthesis model

**Fig 1:**
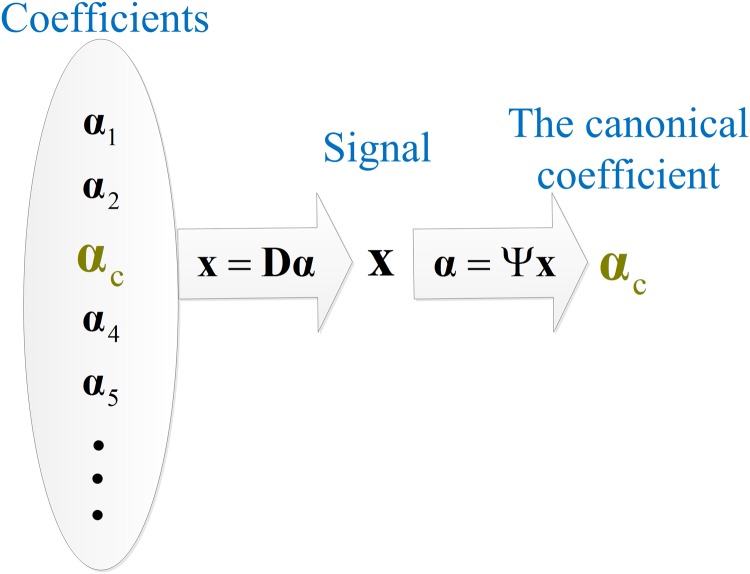
Difference between the coefficients and the canonical coefficient of a signal.

**Fig 2:**
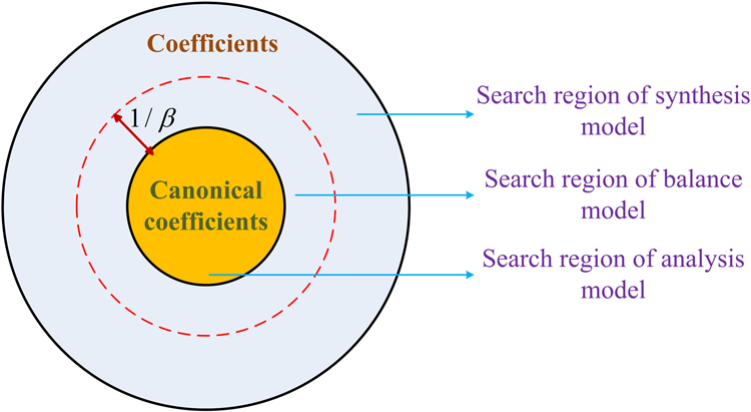
The relation of analysis, synthesis, and balanced models.

For an invertible analysis operator **Ψ**, if we choose the synthesis dictionary as ***D*** = **Ψ** ^−1^, then the analysis and synthesis models are equivalent in the sense that the optimal solutions are the same [35, 36, 38]. However, for a redundant **Ψ**, meaning that ***D*** ≠ **Ψ** ^−1^, these two models are totally different [35]. The difference comes from the fact that a signal can be synthesized from not only one but infinite number of coefficients by a redundant dictionary [36]. However, applying the analysis operator directly to the signal, one can get a unique coefficient called the canonical coefficient denoted as ***α***_*c*_ = **Ψ** ***x*** [51]. In a sense, there is a one-to-one correspondence between the image and its canonical coefficient even in a redundant dictionary. Fig. 1 illustrates the relation between coefficients and the canonical coefficient of a signal. Synthesis model assumes that MR images can be synthesized from sparse coefficients by the dictionary, while analysis model assumes that the canonical coefficients of MR images are sparse. With different assumptions, analysis and synthesis models are searching for solutions in different domains, i.e. the coefficient domain and the canonical coefficient domain, respectively [35]. Fig. 2 provides a visual illustration of this point.

Turning to the performance of these two models, we can not find a unified view in a general case. More researchers prefer to say that these two models fit different types of datasets and it is hard to say which one is better in a general case [25, 35, 36, 52, 53]. Nonetheless, the analysis model is reported to outperform the synthesis model when certain systems are used, and the former is the suggested in these papers.

#### Balanced model

To bridge the gap between analysis and synthesis models, Cai et al. proposed a balanced model [41–44] for tight frame systems. Let **Ψ** and Ψ* be the analysis and synthesis operators associated with a tight frame system. In other words, we have Ψ* **Ψ** = ***I***, and generally **Ψ** Ψ* ≠ ***I***. Then, the balanced model in [41–44] is as follows

$$\begin{array}{c} \hat{x}=\Psi^{*}\hat{\alpha },\;\;\hat{\alpha }=\arg\min_{\alpha }{∥\alpha ∥}_{1}+\frac{\beta }{2}∥\left(I-\Psi \Psi^{*}\right){\alpha ∥}_{2}^{2},\;\text{s.t.}\;{∥y-A\Psi^{*}\alpha ∥}_{2}^{2}\leq \sigma^{2}, \end{array}$$ (6)

where ***A*** stands for a linear degrading operator. Since **Ψ** Ψ* is the projection onto the range of **Ψ**, the term $\frac{\beta }{2}\|(\text{I}-\Psi \;{\Psi \;}^{*})\alpha {\|}_{2}^{2}$ is the squared distance of ***α*** to the range of **Ψ** (or to the canonical frame coefficient), and *β* is a balancing parameter.

When *β* = 0, (6) becomes

$$\begin{array}{c} \hat{x}=\Psi^{*}\hat{\alpha },\;\;\hat{\alpha }=\arg\min_{\alpha }{∥\alpha ∥}_{1},\;\text{s.t.}\;{∥y-A\Psi^{*}\alpha ∥}_{2}^{2}\leq \sigma^{2}, \end{array}$$

which is exactly in the form of synthesis model in (2). When *β* → ∞, (6) imposes that

$$\begin{array}{c} ∥\left(I-\Psi \Psi^{*}\right){\alpha ∥}_{2}^{2}=0\;⟺\;\alpha =\Psi \Psi^{*}\alpha \end{array}$$

which means *α* is a canonical coefficient. Thus, (6) becomes

$$\begin{array}{c} \hat{x}=\Psi^{*}\hat{\alpha },\;\;\hat{\alpha }=\arg\min_{\alpha }∥\Psi \Psi^{*}{\alpha ∥}_{1},\;\text{s.t.}\;{∥y-A\Psi^{*}\alpha ∥}_{2}^{2}\leq \sigma^{2}. \end{array}$$

Letting ***x*** = Ψ* ***α*** leads to

$$\begin{array}{c} \hat{x}=\arg\min_{x}{∥\Psi x∥}_{1},\;\text{s.t.}\;{∥y-Ax∥}_{2}^{2}\leq \sigma^{2}, \end{array}$$

which is exactly in the form of analysis model in (4). Thus, for 0 < *β* < +∞, (6) is a balance between the analysis model and the synthesis model. Fig. 2 presents the relationship of these three models.

### Proposed work

To the best of our knowledge, how the balanced model performs in CS-MRI has never been investigated. More specifically, how the balancing parameter affects the reconstruction is still unknown. Besides, there are also needs to develop an efficient algorithm to solve the balanced model based CS-MRI.

#### Constrained balanced model in tight frame based CS-MRI

Orthogonal wavelets (orthogonal systems) are usually used in traditional compressed sensing MRI [2]. However, orthogonal wavelets may lead to blocky artifacts in image reconstruction [14–17]. On the contrary, redundant wavelets, e.g. shift-invariant wavelets, can significantly improve the image quality [14–17]. Examples of such tight frames are framelet [24], curvelet [20], translation invariant discrete cosine transform [2], and patch-based directional wavlelets [14]. Let **Ψ** ∈ ℂ^*D*×*N*^ with *D* > *N* be the analysis operator of a tight frame, and then its adjoint Ψ* is the associated synthesis operator. The tight frame property implies that Ψ* **Ψ** = ***I***. Because *D* > *N*, the operator **Ψ** Ψ* is not the identity but the orthogonal projector onto the range of **Ψ**. Motivated by the balanced model presented in previous sections, we propose the following constrained balanced model in tight frame based CS-MRI

$$\begin{array}{c} \hat{x}=\Psi^{*}\hat{\alpha },\;\;\hat{\alpha }=\arg\min_{\alpha }{\lambda ∥\alpha ∥}_{1}+\frac{\beta }{2}∥\left(I-\Psi \Psi^{*}\right){\alpha ∥}_{2}^{2},\;\text{s.t.}\;{∥y-UF\Psi^{*}\alpha ∥}_{2}^{2}\leq \sigma^{2}, \end{array}$$ (7)

By tuning the balancing parameter *β*, one has the chance to achieve a balance between the analysis model and the synthesis model.

#### Constrained split augmented Lagrangian shrinkage algorithm for balanced model (C-SALSA-B)

A popular method for solving the analysis model (4) and the synthesis model (2) is the alternating direction method of multipliers (ADMM) [54], which has various origins in imaging sciences and was proposed by several authors independently under different names, e.g., the split Bregman algorithm [36, 55] and the split augmented Lagrangian shrinkage algorithm [56].

When ADMM is applied to solve the minimization arising from the proposed balanced model (7), there are a couple of different formulations available. One formulation is to convert the constraint minimization (7) to an unconstraint one. By Lagrangian multiplier theory, there always exists a positive number *δ* so that (7) is equivalent to an unconstrained minimization

$$\begin{array}{c} \min_{\alpha }{\lambda ∥\alpha ∥}_{1}+\frac{\beta }{2}∥\left(I-\Psi \Psi^{*}\right){\alpha ∥}_{2}^{2}+\frac{\delta }{2}{∥y-UF\Psi^{*}\alpha ∥}_{2}^{2}. \end{array}$$ (8)

By introducing an auxiliary variable ***z*** = ***α***, this minimization is further converted to

$$\begin{array}{c} \min_{\alpha }{\lambda ∥z∥}_{1}+\frac{\beta }{2}∥\left(I-\Psi \Psi^{*}\right){\alpha ∥}_{2}^{2}+\frac{\delta }{2}{∥y-UF\Psi^{*}\alpha ∥}_{2}^{2},\;\text{s.t}\;z=\alpha . \end{array}$$

Then one can apply ADMM to the above minimization to get an approach for solving the balanced model (7). This method was studied in [48] and is referred to ADMM-B throughout this paper. ADMM-B has shown in [48] faster than other algorithms for the balanced model such as the APG method [46] for many digital image processing tasks.

However, it is generally hard to determine the regularization parameter *δ* in (8). Larger or smaller *δ* will cause over or under fitting of the sampled data ***y***. Motivated by this, we propose to, instead of the unconstrained minimization (8), solve the constrained minimization (7) directly. We introduce an auxiliary variable ***z*** = ***α*** and obtain

$$\begin{array}{c} \min_{\alpha }{\lambda ∥z∥}_{1}+\frac{\beta }{2}∥\left(I-\Psi \Psi^{*}\right){\alpha ∥}_{2}^{2},\;\text{s.t}\;{∥y-UF\Psi^{*}\alpha ∥}_{2}^{2}\leq \sigma^{2},\;\;z=\alpha . \end{array}$$ (9)

Following [36, 55–57], we propose to solve (9) by applying ADMM to the following minimization

$$\begin{array}{c} \min_{\alpha }{\lambda ∥z∥}_{1}+\frac{\beta }{2}{∥\left(I-\Psi \Psi^{*}\right)\alpha ∥}_{2}^{2},\;\text{s.t}\;y=UF\Psi^{*}\alpha ,\;\;z=\alpha . \end{array}$$ (10)

with an early stopping criteria $\|\text{y}-\text{U}F{\Psi \;}^{*}\alpha {\|}_{2}^{2}\leq \sigma^{2}$. This method is referred to constrained split augmented Lagrangian shrinkage algorithm for balanced model (C-SALSA-B), following [56]. According to [36, 55–57], C-SALSA-B produces better quality of reconstructed image than ADMM-B. The convergence of C-SALSA-B is studied [36, 57].

More precisely, the augmented Lagrangian of (10) is

$$\begin{array}{c} L_{\mu ,\rho }\left(\alpha ,z,h,d\right)={\lambda ∥z∥}_{1}+\frac{\beta }{2}∥\left(I-\Psi \Psi^{*}\right){\alpha ∥}_{2}^{2}+\frac{\mu }{2}∥UF\Psi^{*}{\alpha -y-h∥}_{2}^{2}+\frac{\rho }{2}{∥\alpha -z-d∥}_{2}^{2}. \end{array}$$

Then, ADMM for solving (10) can be written as

$$\begin{array}{c} \left\{\begin{array}{c} \alpha_{n+1}=\arg\min_{\alpha }L_{\mu ,\rho }\left(\alpha ,z_{n},h_{n},d_{n}\right),\\ z_{n+1}=\arg\min_{\alpha }L_{\mu ,\rho }\left(\alpha_{n+1},z,h_{n},d_{n}\right),\\ h_{n+1}=h_{n}-\delta_{h}\left(\mathrm{UF}\Psi^{*}\alpha_{n+1}-y\right),\\ d_{n+1}=d_{n}-\delta_{d}\left(\alpha_{n+1}-z_{n+1}\right). \end{array}\right. \end{array}$$ (11)

The sub minimization problem w.r.t. ***α*** in the first line of (11) has an analytical unique solution

$$\begin{array}{c} \alpha_{n+1}=\frac{\mu }{\mu +\rho }\Psi F^{*}U^{*}\left(y+h_{n}\right)+\gamma \left(z_{n}+d_{n}\right)+\Psi F^{*}\;\left[\left(1-\gamma \right)I-\frac{\mu }{\mu +\rho }U^{*}U\right]\;F\Psi^{*}\left(z_{n}+d_{n}\right), \end{array}$$ (12)

where

$$\begin{array}{c} \gamma =\frac{\rho }{\rho +\beta }. \end{array}$$ (13)

The proof of (12) is presented in S1 Appendix. When *β* goes from 0 to +∞, *γ* changes from 1 to 0, and the model changes from the synthesis one to the analysis one. The sub minimization problem w.r.t. ***z*** in the second line of (11) is solved by a soft-thresolding

$$\begin{array}{c} z_{n+1}={𝓣}_{\lambda /\rho }\left(\alpha_{n+1}-d_{n}\right), \end{array}$$

where 𝓣_*λ*_(⋅) is the soft-thresholding operator satisfying

$$\begin{array}{c} {𝓣}_{\lambda }\left(x\right)=\max\left\{|x|-\lambda ,0\right\}\cdot \mathrm{sgn}\left(x\right)=\left\{\begin{array}{cc} x+\lambda , & \text{if}\;x\leq -\lambda ,\\ 0, & \text{if}\;x\leq -\lambda <x<\lambda ,\\ x-\lambda , & \text{if}\;x\geq \lambda , \end{array}\right. \end{array}$$

for each entry of ***x***. The proposed algorithm is summarized in Algorithm 1.

**Algorithm 1** C-SALSA-B

**Input: y**, *λ*, *γ*, *ρ*, *μ*, *δ*_*h*_, *δ*_*d*_, ***h***_1_, ***d***_1_, ***z***_1_

  1: *n* = 1

  2: **repeat**

  3: $\alpha_{n+1}=\frac{\mu }{\mu +\rho }\Psi \;{\text{F}}^{*}U^{*}(\text{y}+{\text{h}}_{n})+\gamma ({\text{z}}_{n}+{\text{d}}_{n})+\Psi \;{\text{F}}^{*}\left[(1-\gamma )\text{I}-\frac{\mu }{\mu +\rho }U^{*}U\right]\text{F}{\Psi \;}^{*}({\text{z}}_{n}+{\text{d}}_{n})$

  4: ***z***_*n*+1_ = 𝓣_*λ*/*ρ*_(**α**_*n*+1_ − **d**_*n*_)

  5: ***h***_*n*+1_ = ***h**_*n*_ − *δ*_*h*_(**UF*** Ψ* ***α***_*n*+1_ − **y**)

  6: ***d***_*n*+1_ = ***d***_*n*_ − *δ*_*d*_(**α**_*n*+1_ − **z**_*n*+1_)

  7: *n* = *n*+1

  8: **until** converge

**Output:** $\hat{\text{x}}={\Psi \;}^{*}\alpha_{n}$

## Results

### Experimental setup

**Fig 3:**
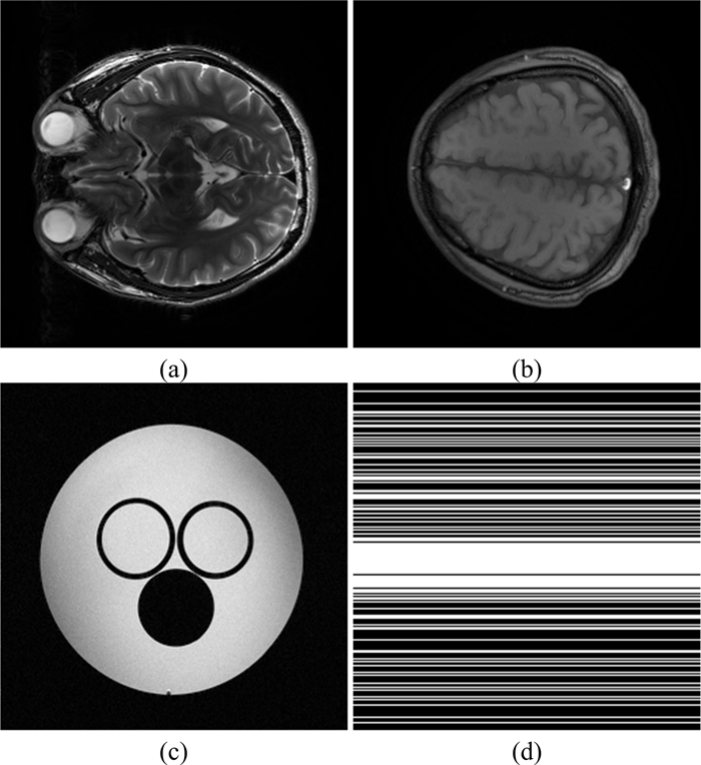
Images used in simulations. (a) is a T2- weighted brain image, (b) is a T1- weighted brain image, (c) is a water phantom image, (d) is a k-space undersampling pattern with 40% data are sampled.

The brain image of size 256 × 256 in Fig. 3 (a) is acquired from a healthy volunteer at a 3T Siemens Trio Tim MRI scanner using the T2-weighted turbo spin echo sequence (TR/TE = 6100/99 ms, FOV = 220 × 220 mm^2^, slice thickness = 3 mm). Fig. 3 (b) is acquired from a healthy volunteer at a 1.5T Philips MRI scanner with sequence parameters (TR/TE = 1700/390 ms with 230 × 230 mm^2^ field of view, 5 mm slice thickness). Fig. 3 (c) is a water phantom image acquired at 7T Varian MRI system (Varian, Palo Alto, CA, USA) with the spin echo sequence (TR/TE = 2000/100 ms, 80 × 80 mm^2^ field of view, and 2 mm slice thickness).

The relative ℓ_2_-norm error (RLNE) defined as

$$\begin{array}{c} \mathrm{RLNE}:=\frac{∥\hat{x}{-x∥}_{2}}{{∥x∥}_{2}} \end{array}$$

is adopted to measure the difference between the reconstructed image $\hat{\text{x}}$ and the fully sampled image ***x*** [14]. Shift-invariant discrete wavelet transform (SIDWT) from Rice Wavelet Toolbox [58] is used as a typical tight frame.

We will compare our proposed C-SALSA-B algorithm with APG [46] and ADMM-B [48]. Parameters for these algorithms are listed in Table 1. These parameters are chosen empirically so that each algorithm reaches the smallest RLNE while maintaining convergence speed as fast as possible. All the experiments are done on a desktop with four Intel Cores i7-2600 CPU at 3.4GHz and 16GB of memory. All CPU time presented in this paper are the average of 5 runs for each experiment.

**Table 1:** Parameters for algorithms used in this paper.

Algorithms	APG	ADMM-B	C-SALSA-B
			*λ* = 0.05
	*λ* = 0.005	*λ* = 0.01	*γ* = 0.5(*β* = 1)
Parameters	*κ* = 1(*β* = 1)	*α* = 0.5(*β* = 1)	*ρ* = 1
	*L* = *κ*+1 = 2	*μ* = 1	*μ* = 1
		*δ*_*d*_ = 1	*δ*_*k*_ = 1
			*δ*_*d*_ = 1

### CS-MRI reconstructions using analysis, synthesis and balanced models

Using the proposed model in (7), one can easily obtain analysis, synthesis, or balanced models by setting the balancing parameter *γ* in (13) to 0, 1 or an arbitrary value in the range (0,1) for CS-MRI. If not specified, the balanced model refers to *γ* = 1/2 without loss of generality throughout this section.

**Fig 4:**
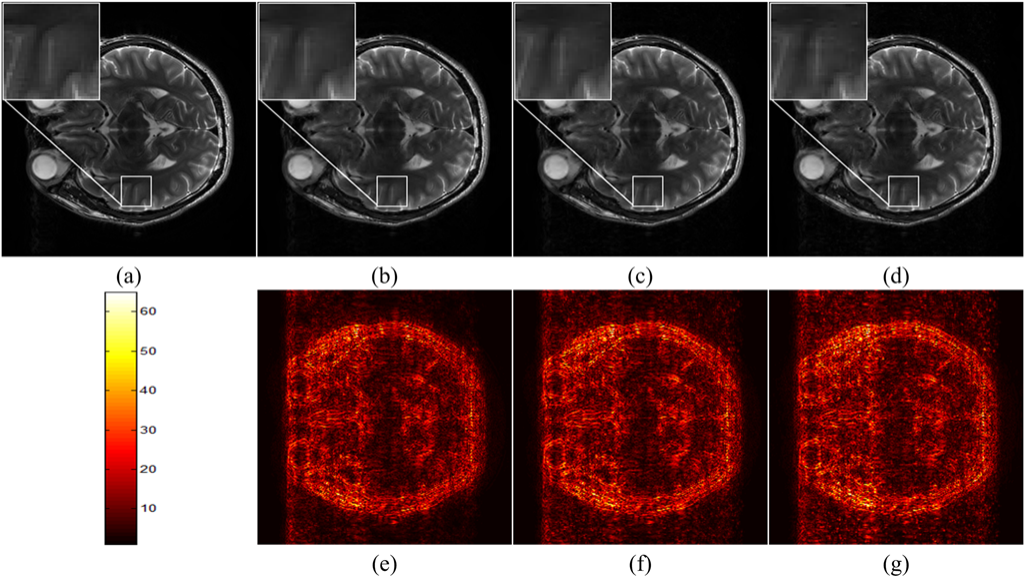
Reconstructed T2 weighted brain images using analysis, balanced and synthesis models. (a) the fully sampled image; (b)-(d) are reconstructed images using analysis, balanced and synthesis models, respectively; (e)-(g) are 6 times scaled reconstruction errors for images in (b)-(d), respectively. The RLNEs for (b)-(d) are 0.114, 0.122 and 0.128.

The simulation results are shown in Fig. 4. We see that the reconstructed image using the balanced model is similar to that using the analysis model, and both of them can remove the artifacts better than using the synthesis model. The reconstruction errors RLNE indicate that the analysis model achieves error slightly smaller than the balanced model. The synthesis model is the worst in this experiment.

### Empirical convergence of C-SALSA-B algorithm

**Fig 5:**
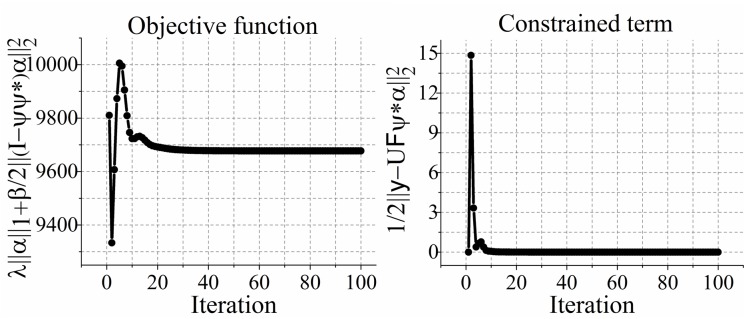
Empirical convergence of C-SALSA-B solving Equation (12). Left is the objective function, right is the value of the constrained term.

**Fig 6:**
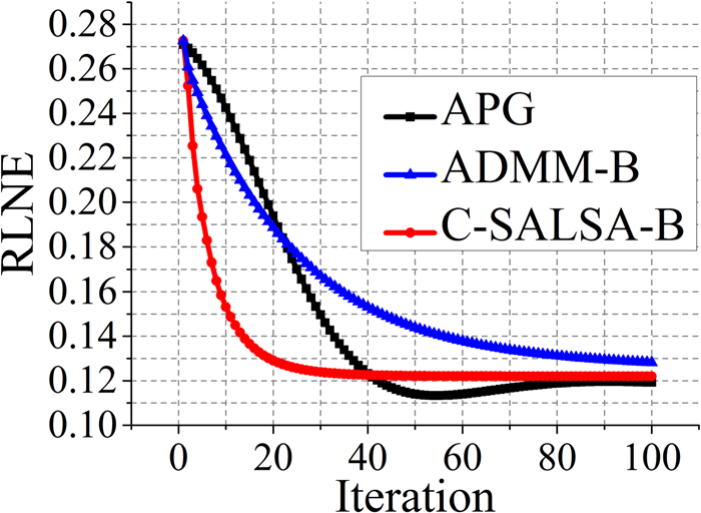
Reconstruction error RLNEs in the iterations using different algorithms.

The convergence curve of the C-SALSA-B is predicted in Fig. 5, and the comparison of the convergence of C-SALSA-B with APG and ADMM-B is plotted in Fig. 6. From Fig. 5, we see that The objective function and the value of the constrained term in (7) approach to a stable state after certain initial iterations, which is consistent to C-SALSA algorithm in [56] (Fig. 4(a)). As shown in Fig. 6, intermediate reconstruction error RLNEs of the proposed C-SALSA-B drops faster than that of APG and ADMM-B. Table 2 shows the computation time of APG, ADMM-B and the proposed C-SALSA-B using T2 weighted brain image dataset in Fig. 3(a). Obviously, the proposed C-SALSA-B algorithm converges faster than the other two algorithms.

**Table 2:** Comparison of different algorithms. The number of iterations in each algorithm is chosen to reach the stable state of RLNE according to Fig. 6.

Algorithm	# of iterations	CPU time in seconds	RLNE
APG	80	16	0.119
ADMM-B	100	13	0.128
C-SALSA-B	30	6	0.123

## Discussion

### Impact of the balancing parameter on reconstructed errors

**Fig 7:**
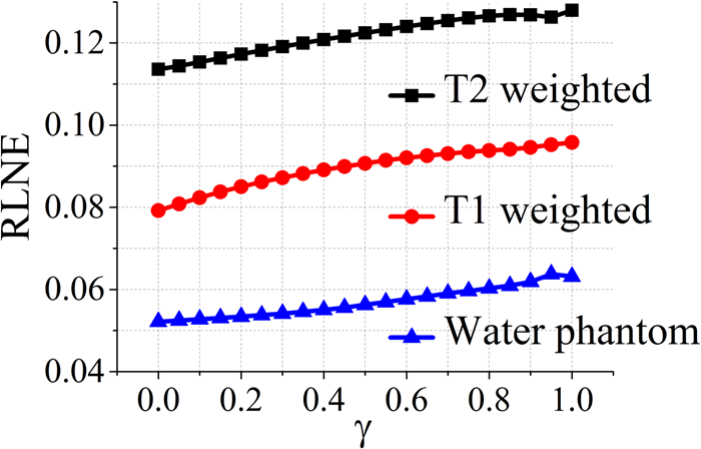
Impact of the balancing parameter *γ* on reconstructed errors for datasets in Fig. 3.

Since the balanced model includes all *γ* ∈ (0,1) in (7), it is necessary to explore the impact of the balancing parameter *γ* defined in (13) on the reconstructed errors. The results are shown in Fig. 7. It implies that RLNE increases monotonically as *γ* goes from 0 (analysis model) to 1 (synthesis model) except one singular point at 0.95.

### Reconstructed errors for different acceleration factors

**Fig 8:**
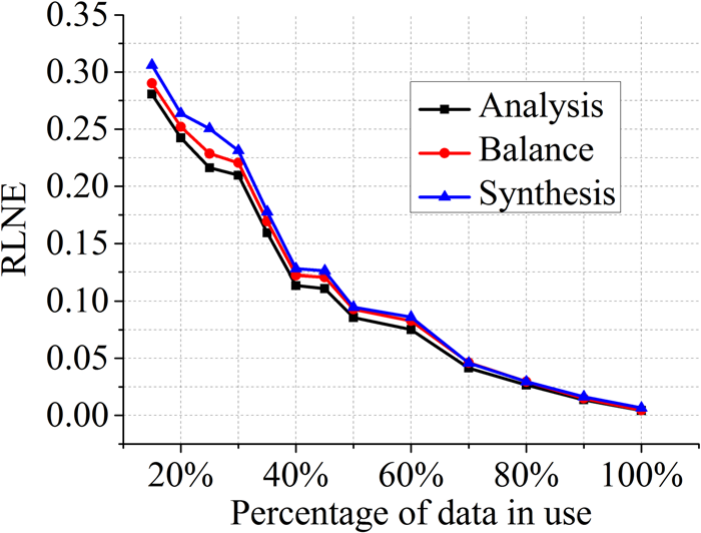
Comparisons of three models for different percentages of acquired k-space data.

We variate the percentage of sampled data goes from 15% to 100% and plot in Fig. 8 the curve of RLNEs by different models against the sampling ratio. We observe that the analysis model always achieve the lowest errors and the synthesis model leads to the highest ones. Reconstruction errors using the balanced model is between other two models.

### Experiments on other tight frames

**Fig 9:**
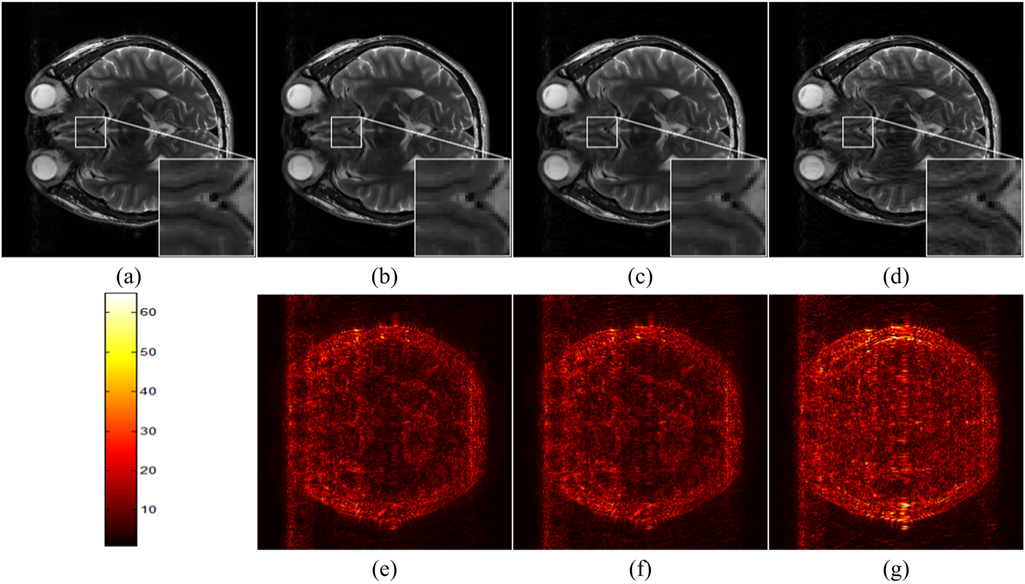
Comparisons on PBDW-based reconstructed images for three models. (a) the fully sampled image; (b)-(d) are reconstructed images using analysis, balanced and synthesis models, respectively; (e)-(g) are 6 times scaled reconstruction errors for images in (b)-(d), respectively. The RLNEs for (b)-(d) are 0.085, 0.086 and 0.114.

**Fig 10:**
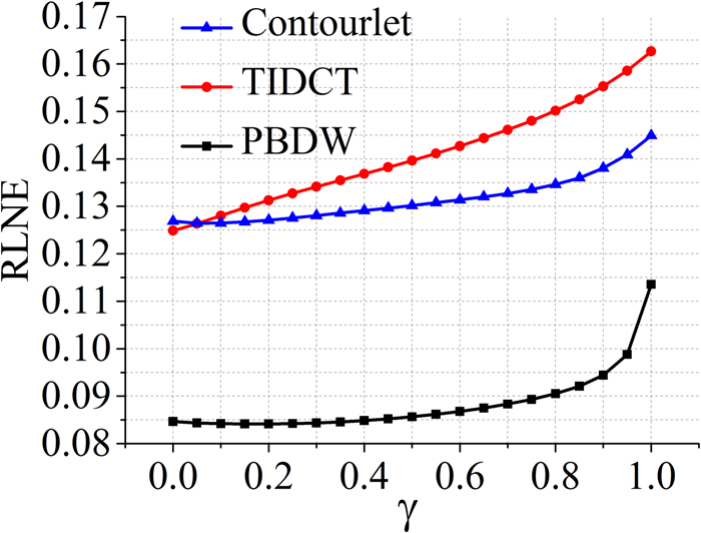
Impact of the balancing parameter *γ* on reconstructed errors when PBDW, contourlets and TIDCT are used as tight frames.

The behaviors of these three models might depend on the tight frame in use. Here, we compare the performance of the three models on a patch-based directional wavelets (PBDW) [14], contourlets [18, 32] and a translation invariant discrete cosine transform (TIDCT) [2]. The reconstructed image using PBDW is shown in Fig. 9. The same phenomenon was observed that reconstructed images using the analysis and balanced models are comparable and both of them contain less artifacts than using the synthesis model. The RLNE criteria also indicates that the analysis model and the balanced model are comparable and these two models achieve lower error than the synthesis model. How the balancing parameter affects the reconstructed errors in PBDW, contourlets and TIDCT are shown in Fig. 10. The trends are similar to that of SIDWT in Fig. 7 but the shapes are a little bit different.

### Comparisons of C-SALSA-B to APG and ADMM-B for more MR images

**Fig 11:**
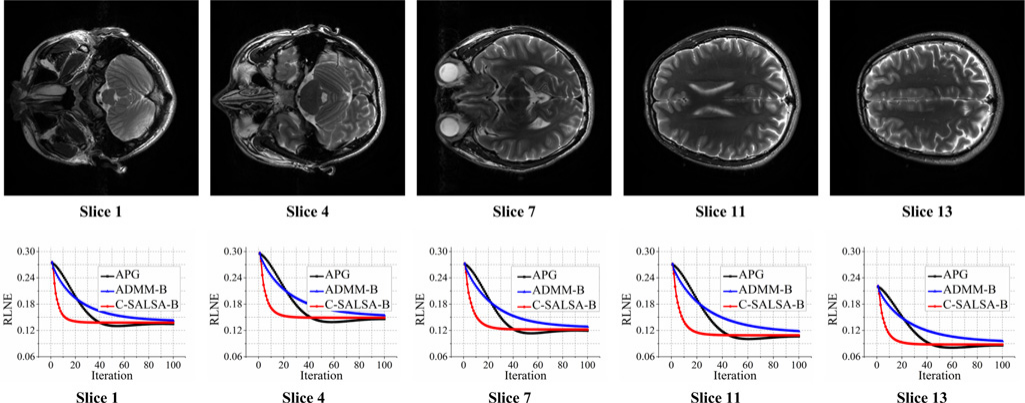
Comparisons of C-SALSA-B to APG and ADMM-B for more MR images.

In this section, we compare our proposed C-SALSA-B to APG and ADMM-B algorithms for more T2 MR images which are different slices of the same dataset as Fig. 3 (a). From Fig. 11, the same phenomenon was observed that the proposed C-SALSA-B converges faster than ADMM-B and APG.

### Comparison of C-SALSA-B to Fast Composite Splitting Algorithm (FCSA)

**Fig 12:**
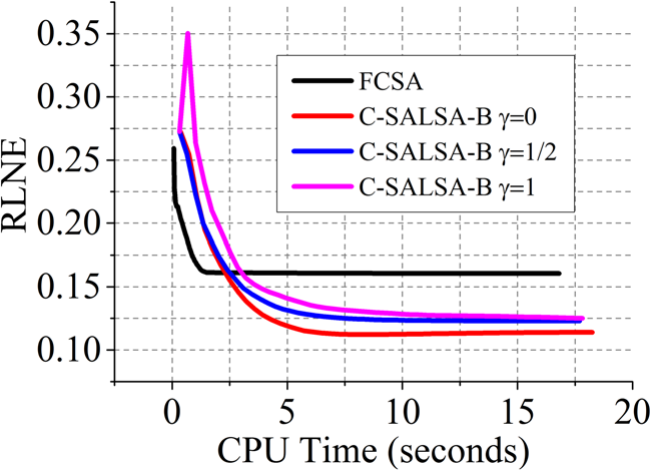
Comparison of FCSA and C-SALSA-B.

In this section, we conduct another experiment to compare our proposed C-SALSA-B algorithm for analysis (*γ* = 0), synthesis (*γ* = 1) and balance (*γ* = 1/2) models to Fast Composite Splitting Algorithm (FCSA) proposed in [13]. Fig. 12 shows that while FCSA converges faster than proposed C-SALSA-B algorithm, C-SALSA-B reaches to the lower RLNE error. Note that the parameter *β* in FCSA has been tuned to be 0.001, which fits for tight frame wavelet and leads to the lowest reconstruction error RLNE. The code of FCSA used in the experiment is downloaded from Dr. Junzhou Huang's website at http://ranger.uta.edu/huang/.

### Experiments on orthogonal wavelets

**Fig 13:**
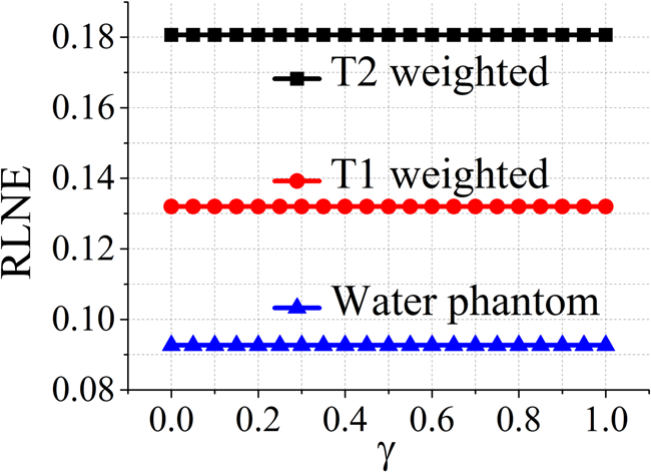
Impact of the balancing parameter *γ* on reconstructed errors when orthogonal wavelets is used.

For an orthogonal transform, the analysis, synthesis and balanced models yield the same results in theory [35]. To testify this, we conduct an experiment for the orthogonal wavelets archived in the Rice Wavelet Toolbox. The result in Fig. 13 shows reconstruction error is not affected by the balancing parameter, indicating the same results are obtained by synthesis, analysis and balanced models.

## Conclusion

A balanced model for tight frame based compressed sensing MRI (CS-MRI) and an efficient numerical algorithm to solve it are proposed in this paper. This new model provides a unified framework to discuss the performance of the analysis and synthesis sparsity models as well as solutions between them. The impact of the balancing parameter on the reconstructed error has been extensively explored. Experiments on magnetic resonance images show that the balanced model can be no better than the analysis model whatever a balancing parameter is optimized. This observation does not change with different forms of tight frame tested in this paper. Results indicate that the analysis model is preferred for tight frames based CS-MRI modelings unless the advantages of the balanced or synthesis model are observed in practice. The proposed C-SALSA-B algorithm is observed to converge faster than typical APG and ADMM-B algorithms in our experiments. However, our tests are limited by certain sparsifying transforms or magnetic resonance images. The power of balanced model for other frames or even other applications needs further investigation.

## Supporting Information

S1 Appendix (PDF)
