# Supplementary material for: Balanced Sparse Model for Tight Frames in Compressed Sensing Magnetic Resonance Imaging
Source: PLoS One. 2015 Apr 7;10(4):e0119584. doi: 10.1371/journal.pone.0119584 (PMC4388626; doi:10.1371/journal.pone.0119584)
Supplement: S1 Appendix — (PDF) [file pone.0119584.s001.pdf]

Appendix A: Proof of (12)

The solution to the minimization in the first line of (11) is obviously given by

$$\boldsymbol{\alpha}_{n+1} = \boldsymbol{A}^{-1}\left[\mu\boldsymbol{\Psi}\boldsymbol{F}^*\boldsymbol{U}^*(\boldsymbol{y}+\boldsymbol{h}_n)+\rho(\boldsymbol{z}_n+\boldsymbol{d}_n)\right],$$

where

$$\boldsymbol{A} = \mu\boldsymbol{\Psi}\boldsymbol{F}^*\boldsymbol{U}^*\boldsymbol{U}\boldsymbol{F}\boldsymbol{\Psi}^* + \beta(\boldsymbol{I}-\boldsymbol{\Psi}\boldsymbol{\Psi}^*)+\rho\boldsymbol{I}. \tag{14}$$

The key step of this proof is to derive the inversion of matrix $\boldsymbol{A}$. We will use the Sherman-Morrison-Woodbury matrix inversion formula [36]

$$(\boldsymbol{X}+\boldsymbol{Y}\boldsymbol{Z})^{-1} = \boldsymbol{X}^{-1} - \boldsymbol{X}^{-1}\boldsymbol{Y}(\boldsymbol{I}+\boldsymbol{Z}\boldsymbol{X}^{-1}\boldsymbol{Y})^{-1}\boldsymbol{Z}\boldsymbol{X}^{-1} \tag{15}$$

provided that $(\boldsymbol{I}+\boldsymbol{Z}\boldsymbol{X}^{-1}\boldsymbol{Y})$ is invertible. We will consider the inverse of the last two terms in (14) as follows

$$\boldsymbol{B} = \beta(\boldsymbol{I}-\boldsymbol{\Psi}\boldsymbol{\Psi}^*)+\rho\boldsymbol{I} = (\beta+\rho)\boldsymbol{I}-\beta\boldsymbol{\Psi}\boldsymbol{\Psi}^*.$$

Using (15) and the property of a tight frame, we get

$$\begin{aligned}\boldsymbol{B}^{-1} = ((\beta+\rho)\boldsymbol{I}-\beta\boldsymbol{\Psi}\boldsymbol{\Psi}^*)^{-1} &= \frac{1}{\beta+\rho}\boldsymbol{I} + \frac{\beta}{\beta+\rho}\boldsymbol{\Psi}\left(\boldsymbol{I}-\boldsymbol{\Psi}^*\frac{\beta}{\beta+\rho}\boldsymbol{\Psi}\right)^{-1}\boldsymbol{\Psi}^*\frac{1}{\beta+\rho} \\ &= \frac{1}{\beta+\rho}\boldsymbol{I} + \frac{\beta}{\rho(\beta+\rho)}\boldsymbol{\Psi}\boldsymbol{\Psi}^*,\end{aligned} \tag{16}$$

where we have used the tight frame property $\boldsymbol{\Psi}^*\boldsymbol{\Psi} = \boldsymbol{I}$. Then, by using the Sherman-Morrison-Woodbury matrix inversion formula (15) again, the inverse of $\boldsymbol{A}$ in (14) is

$$\boldsymbol{A}^{-1} = (\boldsymbol{B}+\mu\boldsymbol{\Psi}\boldsymbol{F}^*\boldsymbol{U}^*\boldsymbol{U}\boldsymbol{F}\boldsymbol{\Psi}^*)^{-1} = \boldsymbol{B}^{-1} - \boldsymbol{B}^{-1}\mu\boldsymbol{\Psi}\boldsymbol{F}^*\boldsymbol{U}^*(\boldsymbol{I}+\boldsymbol{U}\boldsymbol{F}\boldsymbol{\Psi}^*\boldsymbol{B}^{-1}\mu\boldsymbol{\Psi}\boldsymbol{F}^*\boldsymbol{U}^*)^{-1}\boldsymbol{U}\boldsymbol{F}\boldsymbol{\Psi}^*\boldsymbol{B}^{-1}. \tag{17}$$

Moreover, (16) implies

$$(\boldsymbol{I}+\boldsymbol{U}\boldsymbol{F}\boldsymbol{\Psi}^*\boldsymbol{B}^{-1}\mu\boldsymbol{\Psi}\boldsymbol{F}^*\boldsymbol{U}^*)^{-1} = \left(\boldsymbol{I}+\boldsymbol{U}\boldsymbol{F}\boldsymbol{\Psi}^*\left(\frac{1}{\beta+\rho}\boldsymbol{I}+\frac{\beta}{\rho(\beta+\rho)}\boldsymbol{\Psi}\boldsymbol{\Psi}^*\right)\mu\boldsymbol{\Psi}\boldsymbol{F}^*\boldsymbol{U}^*\right)^{-1} = \frac{\rho}{\rho+\mu}\boldsymbol{I}.$$

This together with (17) leads to

$$\begin{aligned}\boldsymbol{A}^{-1} &= \frac{1}{\beta+\rho}\boldsymbol{I} + \frac{\beta}{\rho(\beta+\rho)}\boldsymbol{\Psi}\boldsymbol{\Psi}^* - \frac{\mu}{\rho(\mu+\rho)}\boldsymbol{\Psi}\boldsymbol{F}^*\boldsymbol{U}^*\boldsymbol{U}\boldsymbol{F}\boldsymbol{\Psi}^* \\ &= \frac{1}{\rho}\left[\gamma\boldsymbol{I}+(1-\gamma)\boldsymbol{\Psi}\boldsymbol{\Psi}^* - \frac{\mu}{\mu+\rho}\boldsymbol{\Psi}\boldsymbol{F}^*\boldsymbol{U}^*\boldsymbol{U}\boldsymbol{F}\boldsymbol{\Psi}\right]\end{aligned}$$

where we have used $\gamma = \frac{\rho}{\beta+\rho}$ to the role of the balancing parameter β. Finally, the solution of Eq. (17) is

$$\begin{aligned}\boldsymbol{\alpha}_{n+1} &= \boldsymbol{A}^{-1}\left[\mu\boldsymbol{\Psi}\boldsymbol{F}^*\boldsymbol{U}^*(\boldsymbol{y}+\boldsymbol{h}_n)+\rho(\boldsymbol{z}_n+\boldsymbol{d}_n)\right] \\ &= \frac{1}{\rho}\left[\gamma\boldsymbol{I}+(1-\gamma)\boldsymbol{\Psi}\boldsymbol{\Psi}^* - \frac{\mu}{\mu+\rho}\boldsymbol{\Psi}\boldsymbol{F}^*\boldsymbol{U}^*\boldsymbol{U}\boldsymbol{F}\boldsymbol{\Psi}\right]\left[\mu\boldsymbol{\Psi}\boldsymbol{F}^*\boldsymbol{U}^*(\boldsymbol{y}+\boldsymbol{h}_n)+\rho(\boldsymbol{z}_n+\boldsymbol{d}_n)\right] \\ &= \frac{\mu}{\mu+\rho}\boldsymbol{\Psi}\boldsymbol{F}^*\boldsymbol{U}^*(\boldsymbol{y}+\boldsymbol{h}_n)+\gamma(\boldsymbol{z}_n+\boldsymbol{d}_n)+\boldsymbol{\Psi}\boldsymbol{F}^*\left[(1-\gamma)\boldsymbol{I}-\frac{\mu}{\mu+\rho}\boldsymbol{U}^*\boldsymbol{U}\right]\boldsymbol{F}\boldsymbol{\Psi}^*(\boldsymbol{z}_n+\boldsymbol{d}_n),\end{aligned}$$

which concludes the proof.
